# A fractal approach to dynamic inference and distribution analysis

**DOI:** 10.3389/fphys.2013.00001

**Published:** 2013-01-29

**Authors:** Marieke M. J. W. van Rooij, Bertha A. Nash, Srinivasan Rajaraman, John G. Holden

**Affiliations:** Department of Psychology, CAP Center for Cognition, Action, and Perception, University of CincinnatiCincinnati, OH, USA

**Keywords:** scaling relations, distribution analysis, dynamic systems, cognitive performance, response time distributions, fractal analysis

## Abstract

Event-distributions inform scientists about the variability and dispersion of repeated measurements. This dispersion can be understood from a complex systems perspective, and quantified in terms of fractal geometry. The key premise is that a distribution's shape reveals information about the governing dynamics of the system that gave rise to the distribution. Two categories of characteristic dynamics are distinguished: additive systems governed by component-dominant dynamics and multiplicative or interdependent systems governed by interaction-dominant dynamics. A logic by which systems governed by interaction-dominant dynamics are expected to yield mixtures of lognormal and inverse power-law samples is discussed. These mixtures are described by a so-called cocktail model of response times derived from human cognitive performances. The overarching goals of this article are twofold: First, to offer readers an introduction to this theoretical perspective and second, to offer an overview of the related statistical methods.

Many biological, physiological, and psychological phenomena display time evolving dynamics among their governing processes. Very often these dynamics are straightforwardly observable, as in the back-and-fourth limb movements that are typical of human gait. The most successful and transparent contemporary models of human gait originated in the mathematics of harmonic oscillatory systems such as the driven-pendulum (e.g., Haken et al., 1985; Kugler and Turvey, 1987). The late 15th century research on pendulum behavior was originally motivated by a need for reliable clocks (e.g., Huygens, 1963/1986). The resulting mathematical framework was subsequently adapted to the problem of biological locomotion (among other things). The new application was accommodated by the straightforward observation that, like the pendulum of a clock, both human and animal gaits exhibit relatively regular oscillatory movements (e.g., von Holst, 1939/1973). Clearly, gait's accessibility to measurement facilitated progress in this domain.

Unlike locomotor activity, however, the dynamic evolution of other biological and behavioral systems is, for various reasons, relatively opaque, or simply unobservable. For instance, time evolving dynamics likely support cognitive activity, but those dynamics are more difficult to measure. Worse yet, many stochastic processes entail statistical independence across time. In these cases, scientists may only have access to distributions of measurements that characterize either the same or categorically similar events. They can be utterly disconnected events, related by identity only, not by an obvious adjacent connection in time or space. Thus, a typical outcome measure might index event durations, frequencies, magnitudes, or similar variables. Examples include earthquake magnitudes, computer network traffic, war durations, and countless others. Nevertheless, the shapes of the event-distributions that arise in many systems are often quite lawful. Perhaps the best-known example is when they conform to a Gaussian probability density function.

This article is motivated by the insight that the shape of probability distributions of events reveals information about the dynamics that govern a system's output. The approach leverages the fact that the essential nature of the dynamics that govern many natural *stochastic* systems can be understood without specific knowledge of the components that comprise the system itself (Holden et al., 2009; Holden and Rajaraman, 2012). Inferences about dynamics follow from the statistical behavior of random variables in conjunction with contemporary narratives regarding the behavior of complex systems (Montroll and Shlesinger, 1982; West and Deering, 1995).

To be sure, the methods we describe reveal less complete dynamic information than the methods customarily used in conjunction with observable dynamics, such as phase-space reconstruction. Nevertheless, they do yield enough information to categorize systems in terms of a straightforward taxonomy that distinguishes between *component-dominant dynamics* and *interaction-dominant dynamics*. The event-distributions of component-dominant systems reflect the activity of isolable system components, their time-course, functional details, plus unsystematic additive sources of noise (e.g., Sternberg, 1969; Simon, 1973; Lewontin, 1974). By contrast, the event distributions of interaction-dominant systems reflect emergent, irreducible coordination and coupling among the processes that govern the system (e.g., Pattee et al., 1973; Jensen, 1998). Dynamics are determined by the category of component interactions in the sense that if a given category of system dynamics is in place then particular categories of outcome distributions are a necessary consequence.

Over the course of this article, we present several methods for analyzing and interpreting distributions of observations in terms of their implications for a measured system's dynamic properties. Our entry point is a fractal perspective on distributions that augments the traditional Euclidean geometry that underpins conventional approaches to distribution fitting and parameter estimation. We illustrate how to compute the fractal dimension of an empirical distribution, how to estimate the scaling exponent of an inverse power-law distribution, and finally discuss how to apply maximum likelihood techniques to fit a promising “cocktail” mixture model of response time distributions from cognitive performances.

## Fractal distribution methods

The traditional approach to the characterization of distributions is framed within the context of Euclidian geometry and the standard “signal *plus* noise” theory of measurement error that was largely perfected by the mid-20th century (Stigler, 1986). It is a powerful and useful framework. Arguably, however, many natural systems live on the boundary of its assumptions, or sometimes simply fail to conform to its assumptions. The present goal is to illustrate how distribution analysis can be broadened and enhanced by the inclusion of concepts from fractal geometry. We begin by reviewing the concept of dimension, and generalize the intuitions of the standard Euclidian integer dimension to include the fractal concept of non-integer dimension.

### Fractal dimension

Regular objects, conforming to classical Euclidean geometry, can be characterized by their Euclidean dimension. A punctate observation is a zero-dimensional point; a dimensionless location on a one-dimensional line of measurement. A line is a one-dimensional object; a smooth surface has a dimension of two, and a cube three. Euclidian objects are homogeneous and uniform, breaking them into scaled-down but geometrically identical pieces, reveals their dimension.

**Figure 1:**
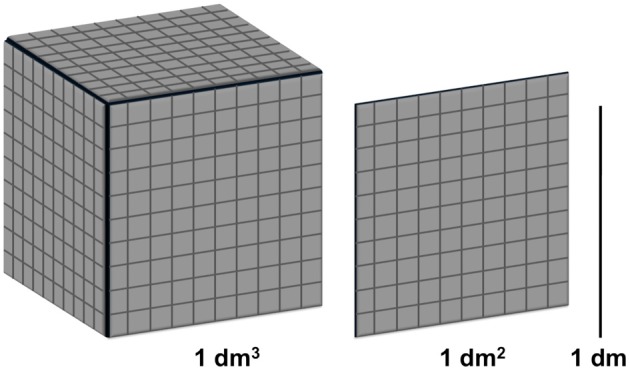
**Depicts the rescaling relationships of a cube, surface, and a line that determine a regular object's Euclidean dimension**.

If the sides of a cubic decimeter are measured in cubic centimeters; that is, if they are scaled down by a factor of 10, then exactly 1000 cubic centimeters will fit in the original cubic decimeter because 1000 = 10^3^. Thus the Euclidean dimension of the original cube is exactly three. In the same vein, if the sides of a squared decimeter are measured in squared centimeters, 100 = 10^2^ squared centimeters fit in the original squared decimeter, and the Euclidean dimension of the original surface is two. Finally, if a line of one decimeter length is measured in centimeters, 10 = 10^1^ centimeters fit in the original line and the Euclidean dimension is one (see Figure 1). This mapping even works for points, 1 = 10^0^, a point cannot be rescaled or divided, and is therefore a zero-dimensional object.

Another way to measure an object's dimension is by determining its topological dimension. Topological dimension in rooted in the idea of connectedness among points in a set. It is computed by determining the dimension of the object required to separate any part of the original set from the rest, plus one. For instance, a line has a topological dimension of one because it can be segmented by a single point that has zero dimension. In fact, regular objects such as curves, surfaces, and solids each have an integer topological dimension of 1, 2, and 3, respectively—values that equal their Euclidean dimension (Bassingthwaighte et al., 1994; Falconer, 2003). Both the Euclidean and the topological dimension take only integer values.

Euclidean geometry, while characteristic of many human artifacts, is an exception to the rule for natural objects. The geometry of most natural objects is highly irregular. Idealized fractal objects are typically comprised of nested copies of the whole object. Their dimension may fall *in between* the integer Euclidean values. The fractal dimension of an object effectively indexes the relative irregularity or space-filling properties of an object. Imagine a piece of thread held taught between two hands, the thread resembles a straight line with Euclidean (and topological) dimension of one. The thread begins to occupy space when it is weaved back and forth, as in a loom, for instance, and the tighter the weave, the more closely it approximates a two dimensional object, cloth. It can be said to “leak” into the next higher, 2nd Euclidean dimension, and thus corresponds to a non-integer *fractal dimension*.

Ours is an admittedly intuitive treatment of fractal dimension. It is a complex mathematical topic and the most formal definition of a fractal concerns a comparison of an object's topological dimension with its space filling properties, as indexed by yet another measure of dimension called the Hausdorff–Besicovitch (H–B) dimension. A set for which the H–B dimension strictly exceeds its topological dimension is a fractal (Mandelbrot, 1977). A more inclusive proposal, also put forward by Benoit Mandelbrot, is that “*a fractal is a shape that is made of parts similar to the whole in some way*” (Feder, 1988).

The take-home point is that objects can be fractal, and are characterized by a non-integer fractal dimension. These facts apply to sets of repeated observations of the self-same process. If repeated measurements of the same object or process always yield exactly the same result, then the measurement converges to a zero-dimensional point—a value commensurate with any observation's Euclidean or topological dimension. However, repeated measurements of natural systems rarely yield sets of identical outcomes. Instead, they almost inevitably vary from observation to observation.

It is this variability or uncertainty in repeated but categorically identical measurements that yields dispersion over the *x*-axis of a dependent measure in an experiment. In this way, the set of measured points begins to “fill” an interval and approximate the one-dimensional *x*-axis of measurement. Just as a more tightly woven piece of thread better approximates a plane, a variable collection of zero-dimensional points roughly approximates a line. Other things equal, such as sample size, a more dispersed distribution will occupy a broader interval and leak further into the domain of the line than a less variable distribution. As such, a distribution's interval-filling properties are indexed by its fractal dimension.

One way to estimate the fractal dimension of a distribution is entailed in the common *relative entropy* statistic. First, generate a histogram of the observations across a fixed interval on the *x*-axis of measurement. The maximum potential range of the observations should define the interval. Once the interval is divided into a convenient number of smaller intervals or bins, the observed frequency in each bin is transformed into a probability, by dividing each bin count by the total number of observations. Next the *Shannon information* (Shannon and Weaver, 1949) is computed across all bins, and divided by the maximum possible entropy—the negative base-2 log of one divided by the total number of bins *B*.

(1) $$FD_{re}=\frac{-\sum p_{i}{\text{log}}_{2}p_{i}}{-{\text{log}}_{2}(1/B)}$$

**Equation 1.** The fractal dimension based on the relative entropy statistic (*FD*_*re*_) as a function of the probability *p*_*i*_ of finding observations in bin *i*, and *B*, the total number of bins.

**Figure 2:**
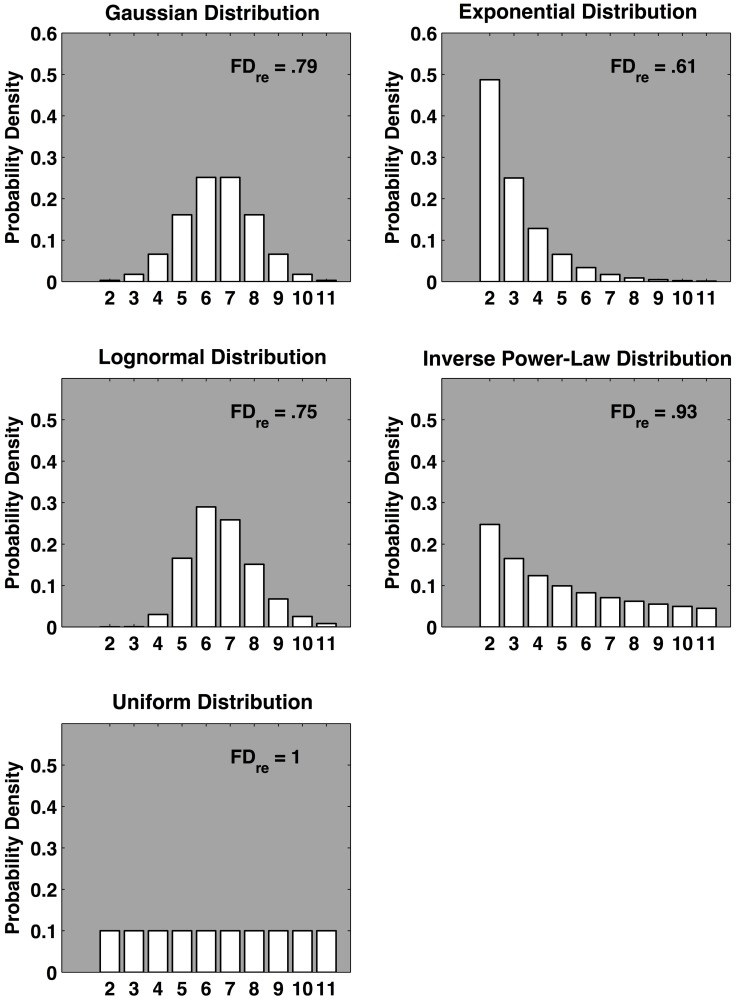
**Five model distributions with approximately equal mean and variance and the corresponding fractal dimension *FD*_*re*_ based on the relative entropy computed using 10 bins.** The top four distributions (Gaussian, exponential, lognormal, and inverse power-law) are ordered according to two broad taxonomies of characteristic system dynamics: component-dominant dynamics and interaction-dominant dynamics (see text for details). The uniform distribution is included to define the upper boundary for *FD*_*re*_. The fractal dimension gauges the relative variability of the respective distributions; the more evenly dispersed, the larger the *FD*_*re*_.

Equation 1 computes the fractal dimension based on the relative entropy statistic, the probability of finding observations in each bin, and the number of bins. The relative entropy statistic measures the relative “evenness” of the distribution; a value of one indicates a uniform distribution where the probability weights evenly cover the measurement interval. Values progressively less than one indicate progressively more “clumpiness” (Seuront, 2010). It can be directly interpreted as a fractal dimension, the degree to which the collection of zero-dimensional points representing the observations leaks into the next higher first Euclidean dimension. Effectively, increases in the variability of the measurements equate with increases in the fractal dimension of the measurements. Figure 2 displays the *FD*_*re*_ and probability densities of four probability distributions that will be discussed in this article, alongside the uniform distribution, which marks the maximum relative entropy, and *FD*_*re*_ of one.

On one hand, using relative entropy as a measure of the fractal dimension is a fairly course grained method for assessing or comparing the dispersion among distributions. Parametric variability statistics are more sensitive. On the other hand, it is largely assumption free. It is most useful for empirical distributions that are not particularly orderly. For instance, distributions that do not appear to conform to a shape that might indicate a standard probability density function could be reasonably adopted as a model. We now consider the more specialized cases where empirical distributions conform to familiar, idealized shapes of defined probability density functions. We provide an introduction to a general taxonomy of random variables that distinguishes the characteristic mode of interactions that give rise to observables. Again, the key focus is characteristic patterns of variability.

## Superposition vs. interdependence

The central theme of statistical physics is that the macroscopic behavior of a system reflects the microscopic arrangements of its constituent parts (Bruce and Wallace, 1989). Characteristic system dynamics originate in the relationships among the processes that comprise a system. Our introduction briefly distinguished two broad taxonomies of characteristic system dynamics: *component-dominant dynamics* and *interaction-dominant dynamics*. They each entail distinct system transactions, superposition, and interdependence, respectively. We now explain how component-dominant dynamics arise.

The term applies to systems that are governed by the activity of largely isolable processes, themselves, their time-course, and their functional details (plus unsystematic noise). Relatively weak interactions among causal processes insure that perturbations affect components locally, unsystematically, and individually. As such, the effects of systematic perturbations can be localized to individual components—that is a consequence and a benefit of encapsulated design. Weak and additive cross-process transactions insure that the *components*, themselves, dominate system output. Systems that express component-dominant dynamics are consistent with Simon's (1973) *nearly decomposable* systems, since they entail minimal linkages across time-scales and minimal within-timescale feedback. Component-dominant dynamics represent a key prerequisite for a successful reductive analysis of a system. They are presumed in the application of standard linear Gaussian statistical techniques such as ANOVA and regression.

### Component-dominant dynamics

The standard *Gaussian* distribution represents an archetypal outcome or end state for systems that are comprised of components whose effects dominate their time evolution. The dispersion or variability around the mean of a Gaussian distribution emerges from the combined, additive influence of innumerable weak, accidental, and mutually independent factors (Gnedenko and Khinchin, 1962; Hays, 1994). Each influence or perturbation affects the outcome, if ever so slightly. Since the factor's effects are independent and unsystematic they cancel each other's influence as often as they reinforce each other, in the long run. Thus, Gaussian distributions emerge from systems whose observables are subject to vast arrays of relatively weak, additive, and independently acting perturbations: component-dominant systems. In effect, the dynamics of superposition simply restate Laplace's Central Limit Theorem. If the assumptions of the theorem are met, then a Gaussian distribution will result. In that case, the distribution's mean is the only real piece of information imparted by the entire distribution.

The standard *exponential* distribution represents a different expression of component-dominant dynamics. Its probability density is *p*(*x*) = (1/λ)*e*^−*x*^ where *x* is the axis of measurement. An exponential distribution often signifies processes that conform to stochastic “counting” or a bottlenecked queuing process. It represents a steady, reliable accrual process that is characterized by the mean (λ) of the distribution. The exponential distribution is thus a typical example of a distribution resulting from a component-dominant process; its properties are fully described by the average rate 1/λ. The exponential is an expression of additive perturbation in time, as the exponential arises when events have a constant average rate per interval of time, and conform to a Poisson distribution, which, in turn, can be approximated by a Gaussian distribution. As with the Gaussian, exponential variability arises from unsystematic additive influences and its mean is the key piece of information imparted by the distribution.

If an exponential rate parameter is sufficient to characterize a process then it could, in principle, be identified and discriminated from other processes with different characteristic rate parameters or distribution functions. System outputs that conform to an exponential support a hypothesis that component processes themselves, dominate a system's transactions and observed variability. Next, we introduce an alternative case, in which the system dynamics are dominated by reciprocal, interdependent, and multiplicative transactions among processes.

### Interaction-dominant dynamics

Understanding how a system's components interact takes priority over identifying the components themselves. This is because one must first determine whether the components can, in principle, be recovered before one goes looking for their signatures in event-distributions, for example (Uttal, 1990; Van Orden et al., 2003, 2005). *Interaction-dominant dynamics* are associated with systems that entail tightly coupled processes spanning a wide range of temporal or spatial scales, including fractal systems. They refer to systems that entail multiplicative and/or interdependent feedback transactions among the processes that govern the system's dynamics. Just as component-dominant dynamics are associated with additivity, and the Gaussian distribution, interaction-dominant dynamics are also consistent with specific categories of distributions.

An *inverse power-law* distribution is a so-called *heavy-tailed* distribution; the heavy tail represents large magnitude, but rare events (Clauset et al., 2009). Thus, it expresses a salient positive skew. If the extreme right tail of an event distribution decays as a power function, then the probability of observing a particular event magnitude, *p*(*x*), is the inverse of the *x* value itself, raised to the *scaling exponent* α (alpha) that is *p*(*x*) ≈ *x*^−α^. The formal mathematical equation of the inverse power-law probability density function is *p*(*x*) = *b* · *x*^−α^, where *b* is a positive constant. The scaling exponent α quantifies the rate of decay of the distribution's tail. In scientific papers, α is normally reported as a positive number, derived from the equivalence of *x*^−α^ and 1/*x*^α^. It is crucial to understand that it indexes a completely different property of data than the scaling exponent α of 1/*f*^α^ or *1/f* noise (e.g., Holden, 2005); the former characterizes the shape of a distribution, the latter describes long-range fractal patterns of correlation across successive observations. They are statistically independent patterns.

Inverse power-law distributions describe phenomena that range from the distribution of online music sales to earthquake magnitudes and citations of scientific publications (Anderson, 2006; Bak, 1996; Redner, 1998). Neurophysiological processes also express power law behavior. For instance, the distribution of endogenous EEG and MEG oscillations are inversely power-law distributed (Linkenkaer-Hansen et al., 2001). Similarly, fMRI measurements of human brains, under untasked conditions, reveal scale-free power-law coordination—correlated relational networks of a given average size, that span approximately three orders of magnitude in their observed frequency (Fraiman et al., 2009). Circular, interdependent feedback transactions likely govern systems that express inverse power-law scaling.

**Figure 3:**
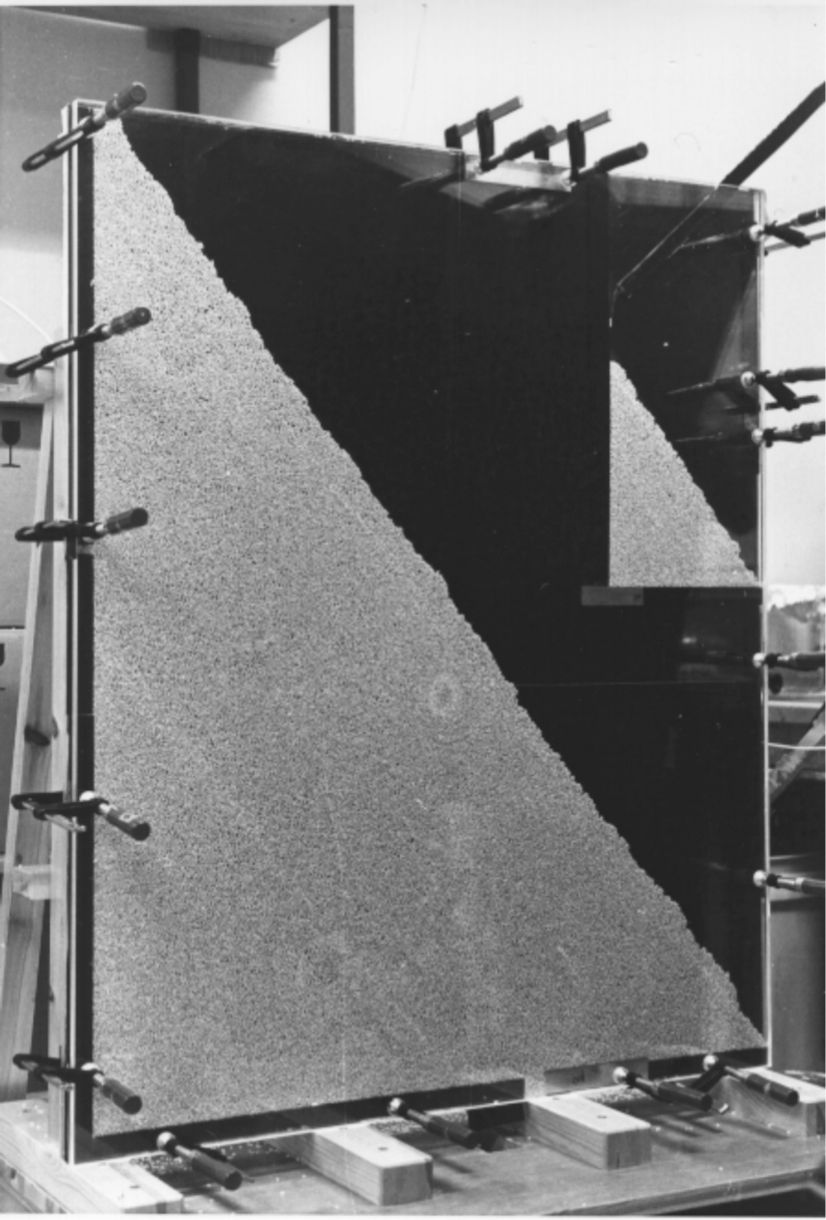
**An example experimental setup used to study the dynamics of one-dimensional rice piles.** The first experimental confirmation that self-organized criticality occurs in granular systems was reported by the Cooperative Phenomena Group at the University of Oslo (Frette et al., 1996). Rice kernels were slowly fed into the pictured device. High-resolution photographs and tracer grains were used to track grain transport. As predicted, the distribution of avalanche magnitudes was consistent with an inverse power-law distribution (Image reprinted with permission from the Cooperative Phenomena Group, University of Oslo).

Power law behavior is symptomatic of self-organizing physical systems poised near a critical point (Bak, 1996; Jensen, 1998). One of several model systems for studying the behavior of self-organized and critical systems is a simple rice pile. Actual rice pile experiments use an apparatus that makes detailed measurements of rice grain activity, as kernels are continuously added to and exit the pile (see Figure 3). Initially, small, localized piles emerge within the larger pile. As the local piles grow, avalanches unfold. At a critical point, a *holistic coordinative balance* emerges throughout the system. The balance is governed by two competing sources of constraint: friction and inertia (Jensen, 1998). From that point on, the rice pile maintains a time-invariant organization, even in the face of the constant perturbation induced by the intermittent clusters of inflowing and avalanching rice. Notably, while the classic lore surrounding this phenomenon concerned *sand* piles, it is in fact long-grain rice rather than sand that entails the proper ratio between friction and inertia to express the characteristic behaviors associated with self-organized criticality (cf. Frette et al., 1996).

When a rice pile is in a critical regime the effects of perturbation are no longer proportional to the size of the perturbation—adding one new grain might result in no change, a tiny avalanche, or a large avalanche, affecting the entire pile. In the long run, small avalanches occur frequently and occasional very large avalanches unfold, all the while the pile maintains a time-invariant average height and slope. An inverse power-law distribution neatly summarizes the relationship between the avalanche magnitudes (indexed by grain counts) and their frequency of occurrence.

More generally, *scale-invariance*, as indicated by power law scaling, is characteristic of many complex systems near a critical point. Scale-invariance may be observed with respect to temporal or spatial variables (or both), but in each case similar changes unfold at all time (or length) scales of the system. Of course, power law scaling alone is not sufficient to establish criticality. For instance, mathematical fractals routinely yield scaling relations, but they are fully deterministic systems of equations. So, while they are iterative systems and exploit feedback, they are not open physical or biological systems. Formally established self-organized critical systems entail nonlinear, far-from equilibrium dynamics, with identified system control parameters that govern qualitative state changes (phase transitions) in the system's observables (order parameters, e.g., see Bruce and Wallace, 1989; Nicolis, 1989). That said, self-organization and critical behavior are generally accepted as plausible working hypotheses with the observation of non-trivial scaling in complex biological systems (e.g., Bak and Paczuski, 1995; Bak, 1996).

A striking outcome of research on critical phenomena is the concept of *universality*—while the physical details of various critical systems vary widely, their behavior near their respective critical points is highly similar. Model rice piles are *dynamic critical phenomena* and express scale invariance and time-invariant organization. *Equilibrium critical phenomena*, such as a superconducting phase transition, arise in certain conductive materials and express scale invariant coordination near the critical temperature at which electrical resistance vanishes in a superconducting phase transition. The physical details of rice piles and superconductive materials could hardly be more distinct. Nevertheless, member systems of both categories of critical phenomena exhibit universalities, such as critical exponents, characteristic fractal dimensions, and scale-free spatial and temporal correlation functions.

By way of summary, the model rice pile system only reaches a critical state when certain grain size and smoothness requirements are met. For instance, if one adds a constraint that changes the balance between inertia and friction so that one or the other term dominates the interactions, the empirical consequences of feedback are minimized, and the rice pile converges on a characteristic relaxation time. Systems in which the effects of feedback are negligible but that are still governed by multiplicative interactions exhibit *lognormal* instead of power law dispersion (Farmer, 1990; Holden and Rajaraman, 2012).

Lognormal distributions are found in various systems in chemistry, biology, ecology, and economics. In biology and ecology, multiplicative processes describe population and organism growth (Preston, 1948, 1962; Koch, 1966; May, 1981; Magurran, 1988). Proportional amplification yields accelerating growth. Thus, Nishiura (2007) discussed the lognormal distribution as a model for the incubation times of viral infections. Similarly, normally distributed economic growth rates yield a lognormal distribution of future investment values because growth operators are multiplicative.

A lognormal distribution arises from pure multiplicative interactions among independent random variables. The Central Limit Theorem established that the sum of many independent random variables yields a Gaussian distribution. A lognormal distribution becomes Gaussian after a logarithmic transform of the measured variable. Summing the logarithms of two or more numbers and then taking the antilog of the sum, yields their cumulative product. This fact offers a route to generalize the Central Limit Theorem to multiplicative interactions among independent random variables. Processes that generate a lognormal distribution directly are analogue to processes that generate a normal distribution. Just as the *sum* of many independent random variables yields a Gaussian distribution, the *product* of many independent random variables yields a lognormal distribution (Koch, 1966; Ulrich and Miller, 1993).

One may envision a loose continuum of ideal distributions spanning the general taxonomy of component-dominant and interaction-dominant dynamics (e.g., Montroll and Shlesinger, 1982; West and Deering, 1995). At one extreme, there is the Gaussian distribution, a signature of weak unsystematic additive interactions among independent, random variables. At the other extreme, there is the heavy-tailed inverse power-law, the signature distribution of interdependent feedback dynamics. The moderately skewed lognormal stands between these two extremes; it arises from multiplicative interactions among independent variables.

**Figure 4:**
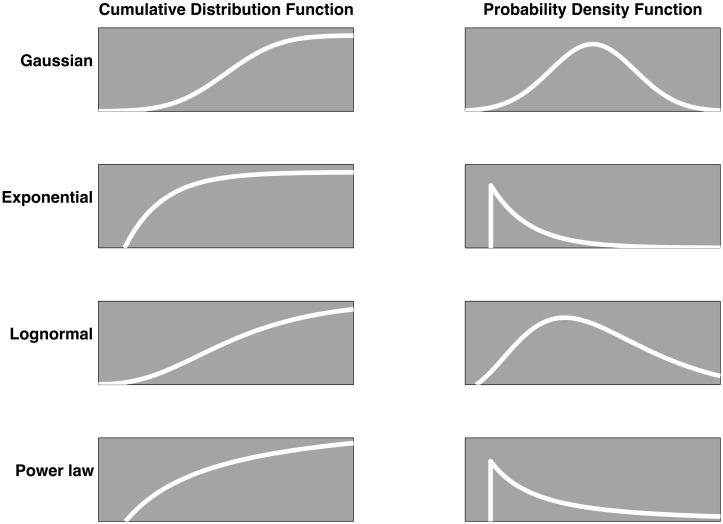
**The plots depict the cumulative distributions (left), and probability density (right) functions of four ideal distributions that signal either component-dominant or interaction-dominant dynamics.** The Gaussian and exponential distributions are symptomatic of component-dominant dynamics while the lognormal and inverse power-law distributions are symptomatic of interaction-dominant dynamics. To the extent that the shapes of *empirical distributions* resemble these various ideal shapes, they likely reveal information about the governing dynamics of the system that gave rise to the distribution.

Admittedly, the distributions we discuss, depicted in Figure 4, represent a tiny subset of the full catalogue of ideal statistical distributions available to scientists. However, no matter their original form, variables conforming to the majority of common statistical distributions are attracted to the Gaussian shape in the case of unsystematic summation, the lognormal in the case of unsystematic multiplication, and the power law in the case of amplification contingent on interdependent feedback operations. Since complex systems likely entail many processes, operating across many time scales, the subset of distributions discussed here represent a plausible entry point for scientific investigation.

We illustrated how the characteristic shapes of ideal distributions supply clues about the dynamics governing a complex system. Dynamics governing a system are determined by the transactions among the processes that compose the system. The shapes of distributions of repeated measurements from a system reveal information about the nature of those transactions. Note that inferences regarding the relation between signature dynamics and a distribution's shape are not necessarily invertible. If the said dynamics govern the interactions of the underlying processes, the various shapes are an unavoidable consequence. However, there are any number of *ad-hoc* ways to contrive the shapes of these distributions. Fortunately, few natural systems represent ad-hoc contrivances.

## Example statistical techniques

This section intersperses example distribution analyses with a bit of practical advice for conducting and using distribution analyses, especially for response time data. We emphasize a complex systems perspective on the phenomena we discuss. We do not claim that a complexity perspective is the only legitimate perspective one could take on these topics. There are, however, many sources that one may consult for conventional narratives on these topics. Complexity theory is a relative newcomer to the physiological, behavioral, and social sciences and offers a promising new perspective on human cognition.

### Histogram methods

This section overviews histogram-based techniques for characterizing power law distributions. The details and relative strengths of these techniques are well characterized in extant references. We strongly encourage readers to consult Newman (2005), Perline (2005), Clauset et al. (2009), and Brown and Liebovitch (2010), for more complete treatments of rank-frequency, histogram, and related methods for characterizing power laws.

**Figure 5:**
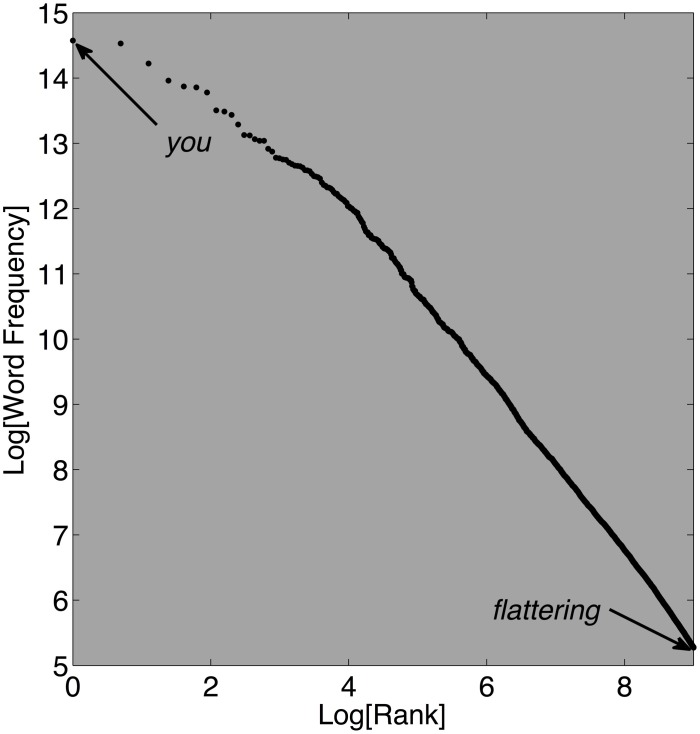
**This plot depicts the Zipf's Law relation between the frequency of occurrence of words in the SUBTLEX_US_ database and the usage rank for approximately 8000 of the most common words (Brysbaert and New, 2009).** The SUBTLEX_US_ database is based on a total of 51 million words that were made available as part of the Elexicon project (http://elexicon.wustl.edu/). Displayed on log-log axes, the rank-frequency relation approximates a straight line, indicating a power law.

The rank-frequency plot is among the earliest techniques routinely used to identify and characterize power law distributions (e.g., Zipf, 1935/1972). The relation between rank and word frequency is the method's namesake, but variables other than frequency can be depicted instead. These plots sort items in terms of their use, or popularity, a *ranking* measure, in conjunction with a measure of *magnitude*. For instance, one could rank items in a retail store in terms of best to worst sellers, and also record their price or how often each item is sold (e.g., see Anderson, 2006). Figure 5 depicts English words with respect to how often they appear in printed text, according to the word frequency counts of Brysbaert and New (2009). The plot illustrates Zipf's Law, an inverse power-law relation between usage rank and frequency of words in written text (Zipf, 1935/1972). The plot's *x*-axis tracks the relative ranking of the words on a logarithmic scale and the *y*-axis similarly represents a logarithmic transform of frequency. When the points in a double-logarithmic rank-frequency plot lie on a straight line, the density is likely a power law (Perline, 2005; Brown and Liebovitch, 2010).

The mathematical properties of logarithms allow a bivariate linear regression analysis to be used to estimate the distribution's scaling exponent. Recall that the general form of the tail of an inverse power-law rank-frequency plot is *p*(*x*) = *bx*^−α^, where *b* is a positive constant and α the scaling exponent. Taking the natural logarithm of both sides of this equation yields *ln*(*p*(*x*)) = *ln*(*b*) − α*ln*(*x*). This denotes a linear relation on double logarithmic axes with slope −α, and scaling exponent α. Thus, a scaling exponent can be roughly estimated from the slope of the distribution's heavy tail on double-logarithmic scales. A fractal dimension *FD*_*rf*_, related but not isomorphic to *FD*_*re*_, can be estimated as *1*/α (Mandelbrot, 1977; Seuront, 2010).

Real languages, whether sampled from specific texts, whole languages, and even translated ancient texts, express Zipf's law (Seuront, 2010). Some authors speculated the pattern is inevitable and claimed it even emerged in randomly assembled letter strings or meaningless text (Miller and Chomsky, 1963). Despite these historical claims, randomly assembled letter strings do not express Zipf's Law. In fact, careful recent simulations and statistical analyses revealed that random texts do not accurately correspond to the expected power law, but real texts do express power laws (Ferrer-i-Cancho and Elvevåg, 2010). Ferrer-i-Cancho and colleagues observed that real texts are constrained by context and meaning, not just by prior character probabilities. They conjectured that the law-like relation between usage rank and frequency results from these competing constraints. Zipf himself speculated that the pattern in language emerges as a consequence of the competing requirements to facilitate a diversity of expressions while preserving simplicity of use. In any case, Zipf's Law appears to reflect the expression of a universal principle of natural language.

Rank-frequency plots are useful tools for computing scaling exponents and estimating a fractal dimension from a distribution of measurements. However, they lack many statistical advantages offered by continuous distribution functions. For example, empty histogram bins become problematic under a logarithmic transform because the log of zero is undefined.

**Figure 6:**
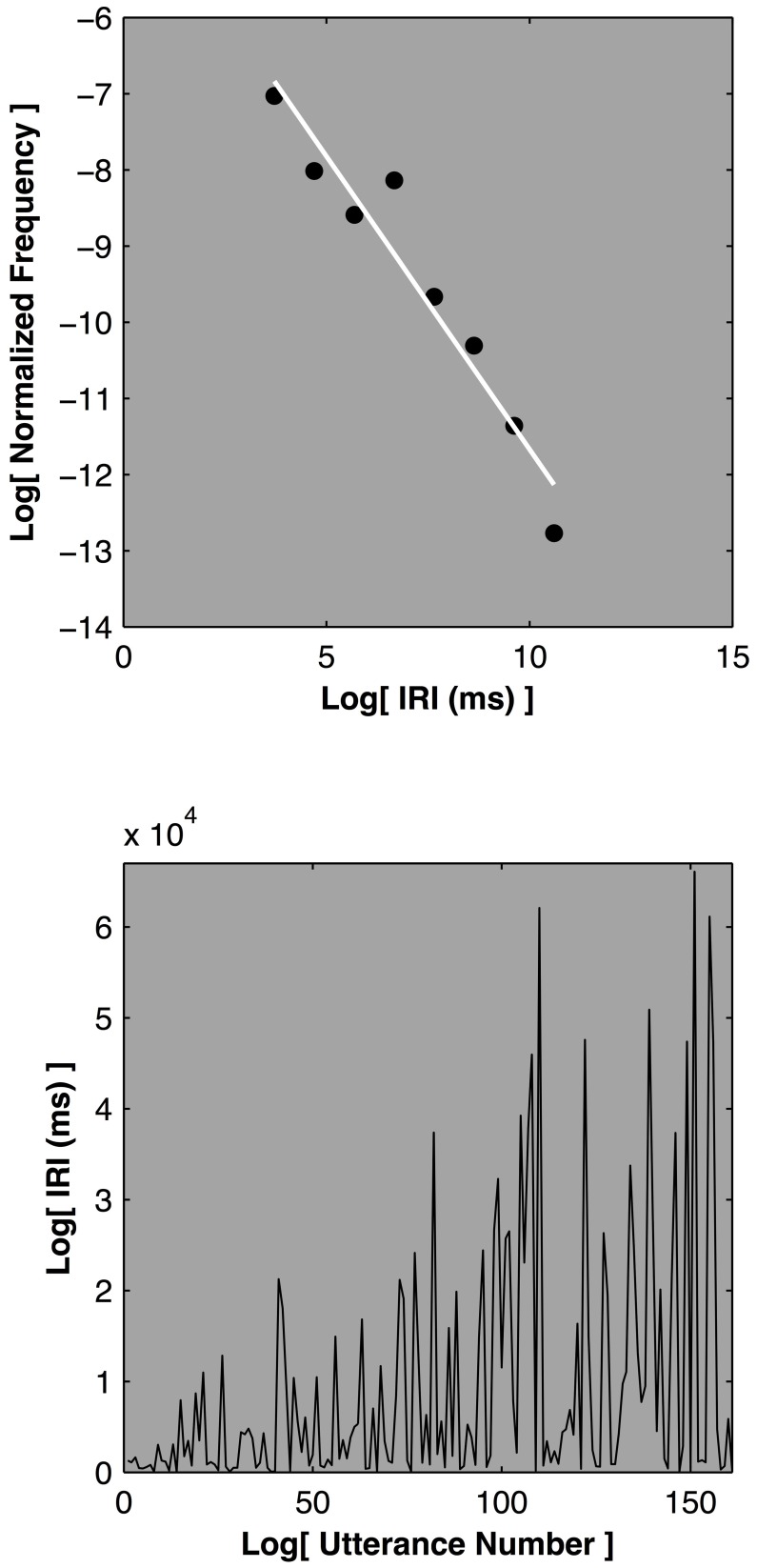
**Depicts the outcome of a free-recall semantic memory session for a single participant from Nash (2012).** The upper plot displays the normalized frequency as a function of the inter-recall interval on log-log axes. The relationship is approximately linear, indicating a power law. The lower plot displays the inter-recall-intervals as a function of the utterance number, the raw data used to generate the histogram.

One way to address the empty bin issue, especially with smaller sample sizes, while maintaining the histogram approach, is to adopt logarithmically spaced histogram bins. Figure 6 depicts the outcome of a free-recall semantic memory experiment (Nash, 2012). Participants were asked to recall as many animals as possible in a 20-min time span. The key dependent measure was the inter-recall-interval (IRI), the elapsed time between the participants successive recall utterances. This paradigm yields data sets that are comprised of perhaps 150–250 observations—a relatively small sample in the domain of power law distributions. Increasing the histogram bin widths logarithmically renders even these relatively small datasets open to statistical characterization (Sims et al., 2007). Measurements can then be characterized and contrasted with alternative distributions, such as an exponential. The IRI distributions are consistent with a power law description and often yield scaling exponent values between 1 and 3 (Rhodes and Turvey, 2007; Nash, 2012). As such they are commensurate with a particular subtype of power law distribution called a Lévy distribution that is implicated in animal foraging activity (Sims et al., 2012, see also Edwards et al., 2012).

### Probability density methods

We now discuss techniques that use kernel density smoothing and maximum likelihood estimation rather than histogram binning and regression fits to characterize empirical distributions. Textbox 1 provides the basics on the Gaussian kernel density estimator; it is a common technique and we adopt it in the examples that follow. The goal is to characterize distributions in terms of standard probability density functions. Our particular focus is on a parametric lognormal and inverse power-law mixture density function, designed to approximate pronunciation and response time distributions that arise from standard laboratory-based cognitive tasks.

Textbox 1 **Characterizing empirical distributions with Gaussian kernel density estimators.**

Kernel density estimation is an empirical distribution smoothing technique. The bars of a histogram are comprised of small rectangular “kernels” that each represents an individual data point. A kernel density uses the same logic, but in place of the standard rectangle, it substitutes a small probability density curve to represent each point. A Gaussian kernel is perhaps the most common kernel, but any continuous and smooth density function can be used. The value of each data point is defined as the mean of each kernel. The standard deviation around the kernel's mean is used for smoothing, it can be set arbitrarily, but is usually set automatically in reference to the variability of the data set. Large kernel standard deviations yield wide kernels and lots of smoothing; small standard deviations yield narrow kernels and very little smoothing. At each point on the *x*-axis the values resulting from the kernel function are summed. Clustered regions of data contribute to larger sums, while sparse regions contribute little to the sums across the *x*-axis. The outcome is then normalized to occupy unit area and yields a continuous and smooth empirical density function. Notably, the density function inherits the properties of the kernels, such as differentiability (see Silverman, 1986; Van Zandt, 2000, 2002). The basic steps for generating a Gaussian kernel density function are as follows:

**Step 1.** Let *x*_1_, *x*_2_,…, *x*_*n*_ be a set of data points perhaps a sample drawn from a population with unknown density *f*. The kernel density estimate, *f-hat* is given by Equation 2, where the kernel, *K*, is the function of a continuous distribution.

(2) $${\hat{f}}_{h}(x)=\frac{1}{nh}\overset{n}{\underset{i=1}{\sum }}K\left(\frac{x-x_{i}}{h}\right)$$

**Equation 2.** Kernel density estimate of a sample *x*_1_, *x*_2_,…, *x*_*n*_, drawn from an unknown distribution *f*.

(3) $$K(x)=\frac{1}{\sqrt{2\pi }}e^{-\frac{1}{2}x^{2}}$$

**Equation 3.** The standard Gaussian probability density function.

**Step 2.** Equation 3 is the standard Gaussian density function, substituting this function for *K* in Equation 2 results in the *Gaussian* kernel density estimator:

(4) $${\hat{f}}_{h}(x)=\frac{1}{nh}\overset{n}{\underset{i=1}{\sum }}\frac{1}{\sqrt{2\pi }}e^{-\frac{1}{2}}{\left(\frac{x-x_{i}}{h}\right)}^{2}$$

**Equation 4.** Gaussian kernel density estimation for a sample *x*_1_, *x*_2_,…, *x*_*n*_, drawn from an unknown distribution *f*. In this equation, the *x* variable refers to the location on the *x*-axis of measurement, and *x*_*i*_ refers to an individual data point and *h* is the smoothing parameter. It is worth noting that *FD*_*re*_ can be computed from a kernel density function. In this case the *B* in Equation 1 is simply the number of points on the *x*-axis for which the kernel density was computed.

Textbox 2 **Transforming Ω_*LN*_ and σ to a linear-unit mean and standard deviation.**

The lognormal mean Ω_*LN*_ and standard deviation σ parameters of the cocktail distribution characterize the lognormal portion of the mixture distribution. They are both defined in natural log units, however. Typically, distributions are characterized by their mean (M) and standard deviation (*SD*) in linear units, and it is useful the transform Ω_*LN*_ and σ values into the linear domain. Equations 5 and 6 specify the relation between the M and *SD* on linear scales and the parameters μ and σ of a lognormal distribution, as specified in the logarithmic domain. They transform the lognormal cocktail parameters into measured units, such as response time. For example, Ω_*LN*_ = 6.2 and σ = 0.15 corresponds to a mean response time of 498 ms (*SD* = 75 ms). Note these values will differ from the empirical mean and standard deviation. Empirical statistics include all the data, and will likely be larger than the Ω_*LN*_, and σ that describe only the lognormal portion of the distribution.

(5) $$M=e^{\mu +\left(\frac{\sigma^{2}}{2}\right)}$$

(6) $$SD=e^{\mu +\left(\frac{\sigma^{2}}{2}\right)}\sqrt{e^{\sigma^{2}}-1}$$

**Equations 5 and 6.** The mean and standard deviation on linear scales, as a function of the logarithmic parameters μ and σ of a lognormal distribution.

As such, our example analyses focus on one particular category of measurements: human response time distributions derived from cognitive tasks. There are many types of response time tasks. Different tasks seek to uncover the functional details of various categories of perceptual and cognitive activities; examples include word recognition, reading, decision-making, perceptual categorization, and many others. Despite this variety of cognitive activity, most tasks similarly impose discrete trials, and each trial presents a single stimulus. Participants are timed as they perform each elementary cognitive act. Once they respond, often with a button press signaling a specific response, the timer stops. Thus, response time is the interval of time that elapses between the onset of a stimulus and the collection of a response in a laboratory-based cognitive task.

We focus on response time data from a mental rotation task. On each trial of the task, a single character from the set 2, 5, 7, G, J, and R was presented. The characters' rotation ranged from 0° to 180° in 60° increments. The stimuli were presented in random order, on half the trials in a normal orientation, and on the other half, mirror-reversed. Participants pressed one key if the character was presented in its normal orientation and another when mirror-reversed, as quickly and accurately as possible.

Broadly speaking, for this and related paradigms, statistical analyses reveal approximately constant increases in mean response time, as a function of both the rotation and orientation factors. This outcome was originally put forward as evidence that an analogue cognitive process literally rotates a mental representation of each character back to the normal orientation, at a constant rate, to accomplish the orientation judgment (e.g., Cooper and Shepard, 1973; Cooper, 1975; Shepard and Metzler, 1988).

Next, we present novel analyses, conducted on a subset of response time data collected as part of a Master's thesis project (Ruzicka, 2005). Figure 9 depicts kernel smoothed response time probability densities for the normally oriented, 60° and 120° rotated characters as straight, dashes and dotted black lines, respectively. The plotted distributions represent correct individual response times, aggregated across 17 of 27 total participants. The 17 participants were selected because they each achieved overall error rates of 10% or less. The density function shapes make it clear that mean response time increases as a function of rotation. However, the density functions express complex changes in shape: increasing rotation results in more variable and skewed distributions, as if they were progressively stretched. Now we introduce a distribution function that describes these response time distributions in terms of a probabilistic mixture of lognormal and power law samples.

### The cocktail model

The cocktail model was originally conceived as a description of individual participant's pronunciation times derived from the speeded naming task (see Holden et al., 2009; Holden and Rajaraman, 2012). Pronunciation time is the elapsed time required to begin speaking a word into a microphone, once a printed target word is presented on a computer screen in a speeded naming task. As such, pronunciation time is a subtype of response time.

Stochastic systems yield distributions of measurements and in any reasonably complex biological system innumerable immediate and historical constraints attenuate measurement variability. In a cognitive act, constraints arise from a participant's idiosyncratic personal history, their present state of body and mind, and task-imposed (environmental) constraints (Hollis et al., 2009; Van Orden et al., 2012). On any given trial in an experiment the laboratory protocol delineates task constraints, but a vast array of additional idiosyncratic constraints are also sampled. Relevant constraints serve to cohere and stabilize a given cognitive activity. Most important, if the system is governed by interaction-dominant dynamics, at minimum, probabilistically sampled constraints are expected to influence the observable multiplicatively, yielding lognormal behavior. Competing constraints or the absence of sufficient constraints may amplify variability in interdependent feedback dynamics, yielding power law behavior. The end result is likely to be a mixture of samples that indicate a continuum of relative dynamic stability.

Since lognormal patterns of variability arise from relatively homogenous multiplicative interactions, lognormal samples represent more stable interactions among the processes and constraints governing a given act. By contrast, power law distributions emerge in the context of more balanced competition among constraints, or more weakly constrained transactions among governing processes. For instance, interdependence and power law behavior is associated with highly context sensitive near-critical physical systems. The cocktail model attempts to capture this continuum as straightforward mixtures of lognormal and inverse power-law samples. Thus, for any given fit to empirical data, the lognormal and power law samples are mixed in fixed proportions, just as the various liquids in a cocktail are mixed in fixed proportions.

The shape and location of the cocktail distribution are controlled by four free parameters: a lognormal *mean* and *standard deviation* (Ω_*LN*_ and σ), a power law *scaling exponent* (α) and a *power law weight* parameter (ρ_*PL*_), see Table 1. Three additional parameters refer to the relative proportions of lognormal samples in the front and back-end of the distribution (ρ_*FLN*_ and ρ_*BLN*_), and the onset threshold of the power law (Ω_*PL*_). Their values, however, are fully determined by the free parameters to insure a smooth, continuous, and legitimate density and distribution function. Some of the relationships among the cocktail model parameters are described in Table 1. Additional details regarding the model's parameters and its full derivation can be found in Holden and Rajaraman (2012).

**Table 1:** **Parameters of the cocktail distribution**.

**Parameter**	**Description**	**Details**
Ω_*LN*_	The mean of the lognormal portion of the cocktail mixture distribution.	Ω_*LN*_ tracks the location of the lognormal portion of the cocktail distribution along the *x*-axis of measurement. It is expressed in natural-log units. (See details in Textbox 2 on transformation to linear units).
σ	The standard deviation of the lognormal portion of the cocktail distribution.	σ describes the dispersion of the lognormal portion of the cocktail mixture distribution and is depicted on a natural-log scale (see also Textbox 2).
α	The scaling exponent of the inverse power-law portion of the cocktail distribution.	α characterizes the dispersion of the power law portion of the cocktail distribution. It describes the decay in the slow tail of the distribution. Plausible values of α range from 1 to about 10, values outside this range are suspect, and likely indicate a poor fit.
ρ_*PL*_	The relative weight of the power law distribution in the tail of the cocktail distribution.	ρ_*PL*_ indicates the portion of the mixture attributed to the power law portion of the cocktail distribution. ρ_*FLN*_, ρ_*BLN*_ together indicate the portion of the distribution attributable to the lognormal. ρ_*FLN*_ corresponds to the portion of the lognormal that falls to the left of Ω_*LN*_ and ρ_*BLN*_ captures the portion right of the Ω_*LN*_. All together, the three portions must sum to 1, the area under the density curve.
ρ_*FLN*_, ρ_*BLN*_, Ω_*PL*_	The relative weight proportions of the lognormal distribution in the front (FLN) and back end (BLN) of the distribution, and the onset threshold of the power law.	The values of these three parameters are constrained by the values of the four free parameters to ensure a smooth and continuous legitimate probability density function.

**Table 2:** **Cocktail parameters corresponding to the three example empirical response time distributions taken from a letter rotation task in Figure 7**.

	**Ω_*LN*_**	**σ**	**α**	**ρ_*PL*_**	**Ω_*PL*_**	***p*-value**
Normal Rotation	6.44	0.14	3.35	0.56	657	0.27
60 Degrees	6.50	0.15	3.21	0.55	702	0.39
120 Degrees	6.62	0.16	3.29	0.61	793	0.24

The lognormal location parameter, (Ω_LN_) was used to capture the bulk of the observed shape changes across the three conditions, in a rescaling test. Goodness of fit was computed using the 2-step bootstrap procedure described in Textbox 3.

There are several ways to approximate or fit a model distribution to an empirical distribution. For instance, one could compute a nonparametric Gaussian kernel estimate of the sample distribution and then use non-linear least squares to approximate the distribution's parameters. A more common approach is to use search algorithms that compute maximum likelihood estimates of the model's parameters. Van Zandt (2000; 2002) provides an accessible introduction to both the methods and the statistical properties of a number of standard response time models.

The goal of maximum likelihood estimation is to adjust a model's parameters, such as the cocktail distribution, so that the overall probabilities under the density curve are maximized. The essentials of the algorithm are straightforward. First, a guess is made for each parameter. There are numerous ways to make an initial guess, ranging from “eyeballing” the distribution to generating quantitative estimates based on special transformations of empirical statistics. Next, the probability density is computed at each point on the *x*-axis of measurement representing all observations. A point-estimate of the probability is returned for each observation. The sum of the natural logarithm of each probability is computed, yielding a summed log-likelihood value. The bigger this number, the more likely it is to observe the sample, given the model and its specific parameter settings. Computerized search algorithms are then used to iteratively explore the parameter space for even larger log-likelihood values, until an apparent maximum value is reached. The search stage of the process represents an entire statistical sub-discipline, and we do not discuss it here (see Press et al., 1992). Some search algorithms, instead of maximizing the summed log-likelihood, minimize the negative summed log-likelihood. Matlab scripts that accomplish this procedure for the cocktail model can be downloaded from: http://homepages.uc.edu/~holdenjn/.

**Figure 7:**
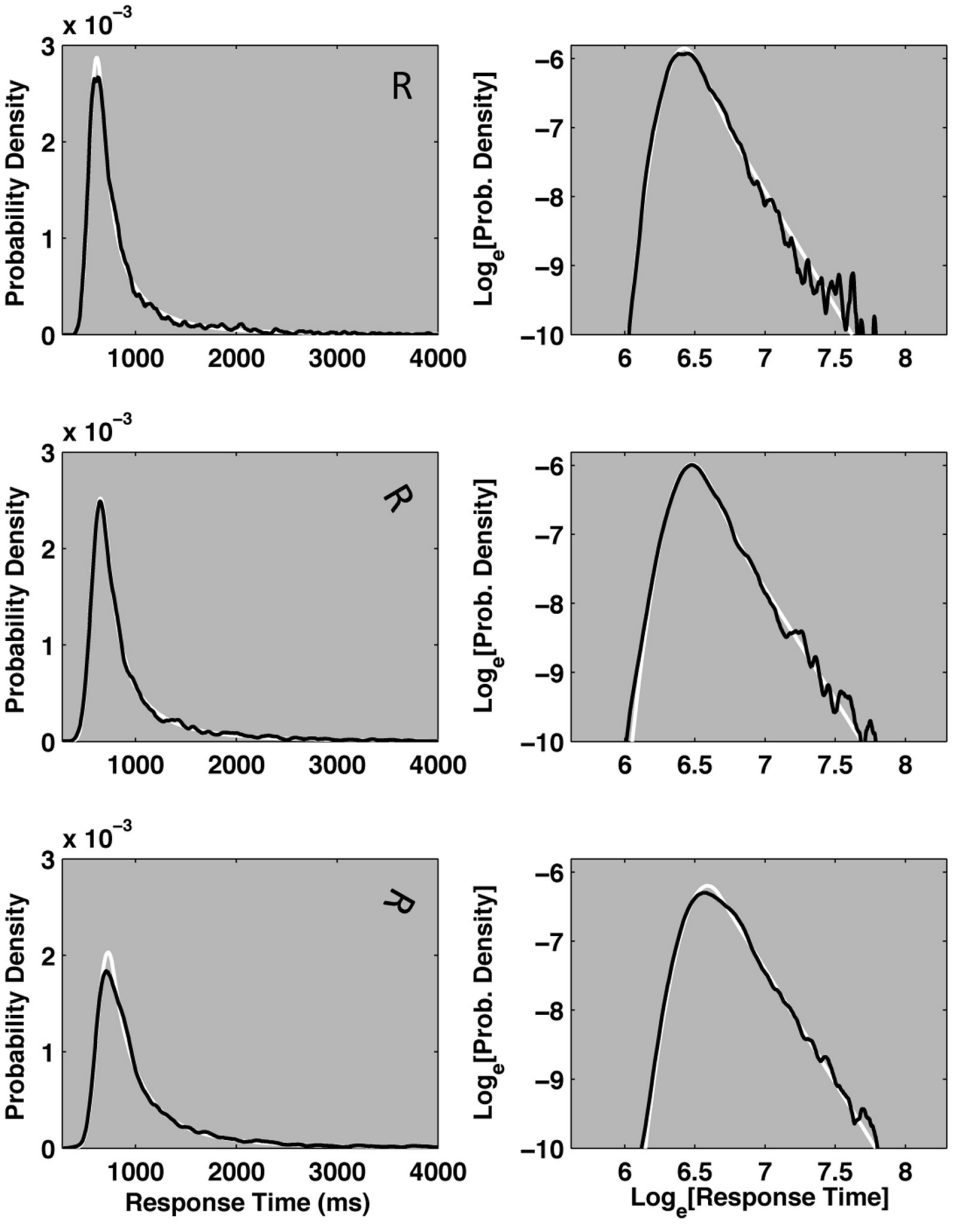
**The plots depict three example empirical response time distributions taken from a mental rotation task presented in Ruzicka (2005).** Each plot depicts the probability density function of an empirical pronunciation time distribution aggregated over 17 participants. In each plot, the black line represents a kernel-smoothed empirical probability density function. Maximum likelihood fits of the Cocktail model are depicted in white, behind the empirical density functions. The three left-hand plots represent the response time distributions for normally oriented and 0°, 60°, and 120° rotated characters on linear axes. The three right-hand plots depict the same empirical and ideal cocktail distributions on double logarithmic axes, and make the power law decay of the distributions' tails more apparent. All three conditions can be reasonably approximated by the cocktail distribution.

The left column of plots in Figure 7 display kernel density estimates of the same empirical mental rotation distributions depicted in Figure 9, now as solid black lines on three separate plots. Maximum likelihood fits of the cocktail mixture are depicted as white lines plotted behind the empirical density functions. The model reasonably captures the empirical distributions. All three distributions generated reliable fits (based on the 2-step bootstrapped K–S test described in Textbox 3). Given that the cocktail model was developed to describe the shapes of individual participant's pronunciation time distributions, its apparent success at describing response time distributions aggregated across different individuals is encouraging.

Textbox 3 **Goodness of fit.**

The cocktail distribution-fitting code returns four free cocktail parameters and three additional determined parameters. However, goodness of fit must then be assessed in some manner. There are many procedures available to complete these tests. One technique is the so-called Kolmogorov–Smirnov test for comparing a sample distribution with a reference probability distribution. For each point on the *x*-axis, the difference (*D*) is computed as the absolute value of the difference between the empirical and the model distribution. The *maximum* of those differences is the *D* statistic. If the best-fitting parameters were used to define the model distribution, then a Monte Carlo technique is recommended to evaluate the plausibility of the fit. Clauset et al. (2009), described one such method: First, a synthetic dataset is generated using the best-fit model parameters. Second, the synthetic dataset is itself fitted with the model, and then *D* is computed with respect to the synthetic dataset and its own best-fit parameters. The resulting *D* value is then retained. This 3-step procedure is repeated 2500 times, resulting in a distribution of 2500 *D* values. Significance (a *p*-value) is computed as the proportion of synthetic datasets with *D* larger than the *D* resulting from a contrast to the empirical dataset and its own best-fit model. If the significance value is *small* (e.g., *p* < 0.1), few synthetic datasets yielded a larger *D* than the empirical dataset, and the empirical distribution *is not* likely a member of the population described by the model. If the significance value is *large* (e.g., *p* > 0.1), many synthetic datasets yielded a larger *D* than the empirical dataset and the empirical distribution is a plausible, but not necessary candidate member of the population described by the model.

This resampling procedure is very sensitive, and one must carefully evaluate the impact of routine statistical procedures and other artifacts, such as data censoring and measurement noise, on the outcome of any goodness of fit procedure. For example, simulations that added Gaussian noise with *SD* equal to 1% (5 ms) of the average variability of a true synthetic cocktail distribution revealed that the Clauset et al. (2009) 3-step Monte Carlo method ruled out the cocktail as a plausible model 66% of the time. By contrast, a 2-step version of the procedure, that omitted a best-fit of the synthetic data (step 2), ruled out the cocktail model as plausible 20% of the time with the addition of 5% (32 ms) Gaussian noise. Cognitive activity is known to entail intrinsic and extrinsic sources of noise (Diependaele et al., 2012). On the other hand, the 2-step procedure is recognized as biased in favor of a fitted model, relative to the Clauset et al. 3-step approach. One potential safeguard is to focus on relative goodness of fit judgments, by using identical techniques on a few candidate models. Then each model is subject to the same procedures. Statistical mimicking and over-fitting are long recognized issues in the modeling literature and, so far, no one-size-fits-all solution has emerged. Nevertheless, this issue can be ameliorated somewhat by focusing on candidate models that are motivated theoretically and corroborated by independent sources of evidence (Van Zandt and Ratcliff, 1995).

Nevertheless, an aggregation approach requires that individuals contribute relatively homogeneous distributions to the aggregate or omnibus distribution. Otherwise, one risks either successfully fitting a statistical artifact, a set of individual distributions that are not individually consistent with cocktail mixtures, but when combined appear as such. The alternative risk is unsuccessfully approximating an idiosyncratic aggregate of distributions despite the fact that individually, they can be legitimately described as cocktail mixtures. For instance, when the participants that generated error rates greater than 10% were included in the aggregate distribution, the cocktail model failed to fit all but the 0° condition. Quite often, there are many ways to perform a task poorly, and very few ways to perform it well. Thus, including all the participants' responses in the empirical distribution likely introduced multiple categories of performance, and the omnibus distribution became too heterogeneous to be successfully approximated by a single cocktail distribution. Similarly, neither of the two aggregate 180° distributions (normal and mirror-reversed) was successfully approximated by the cocktail model. Naturally, these potential pitfalls apply to all model distributions, not just the cocktail distribution (Estes and Maddox, 2005).

In any case, the cocktail distribution is a statistically reasonable description of the three example rotation response time distributions. Examining how the cocktail parameters tend to change across conditions offers insight into how a given manipulation affects performance dynamics. For instance, if the power law proportion increases at the expense of the lognormal proportions, then the manipulation plausibly increases the likelihood of interdependent dynamics. Conversely, if the proportion parameters controlling the power law tend to decrease, and/or the alpha parameter increases, the manipulation may stabilize cognitive dynamics.

Of course, more complex and idiosyncratic patterns of change are possible as well. Several parameters might change as a function of differences across individuals or across conditions. Effectively, the cocktail parameters fall into two broad categories: parameters that control location (Ω_*LN*_ and Ω_*PL*_) and parameters that control variability and skew (σ, α, ρ_*FLN*_, ρ_*BLN*_, and ρ_*PL*_). This is important to keep in mind when interpreting parameter changes. Occasionally, a fitting operation will return an extremely large power law threshold (Ω_*PL*_) or scaling exponent (α). A large discrepancy between this threshold and the lognormal mean may indicate a gap in the empirical distribution, possibly resulting in a spurious local likelihood minimum. Similarly, scaling exponent values greater than 10 or so are an indication that the power law is likely superfluous to the fit. In that case, a pure lognormal or another model may be more appropriate. Excepting wishful thinking, we know of no viable rationale that identifies the model's individual parameters with specific cognitive functions or activities.

It is important to recognize that the cocktail model is descriptive, and that it relies on a reverse inference regarding the relation between dynamics and their expression in measurements. This reverse inference is common in scientific enterprises, an identical logic yields the routine conclusion that if a Gaussian is observed, the system's dynamics are additive. Given that scientists lack *a-priori* knowledge about how any given cognitive manipulation actually impacts neurophysiological dynamics, there really is no guarantee that one can make sense of observed parameter changes for the cocktail model, or other models.

### Rescaling

One specific empirical pattern the cocktail model is capable of elucidating is a rescaling relation. All the location and variability parameters are defined in the logarithmic domain (an exception is the power law threshold, but one can simply compute its natural log for a rescaling test). Rescaling is indicated if location changes, in the logarithmic domain, are the only reliable differences that appear among the model's parameters in contrasts across a given set of conditions. These contrasts can be conducted with the help of bootstrap resampling techniques (Efron and Tibshirani, 1991).

**Figure 8:**
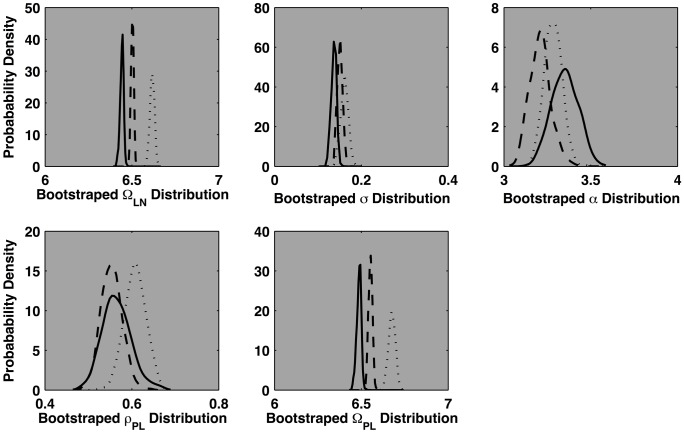
**Each plot depicts the bootstrapped distribution for each of five parameters of the cocktail model.** The outcomes for the 0°, 60°, and 120° conditions are depicted as solid, dashed, and dotted lines, respectively. The bootstrapping procedure randomly resamples the empirical response time distributions 300 times, with replacement. The model is fit to each resampled data set and the resulting distribution of parameter values for each of the three mental rotation conditions are depicted in plots. Identical analyses of the mirror-reversed conditions indicated the 120° distribution as a rescaled version of the 0° and 60° distribution which, themselves were nearly identical.

Figure 8 depicts the outcome of a rescaling test completed for the normally oriented 0°, 60°, and 120° rotations. Each density function represents 300 bootstrapped (resampled) replications of the cocktail fit. The bootstrapped parameter distributions can be treated as standard errors for each corresponding parameter. Parameter distributions that overlap within each other's lower 2.5 and upper 97.5 percentiles are not likely different, distributions that are segregated beyond these thresholds are likely different. The plots for each parameter illustrate that only the lognormal mean and the power law threshold are reliably segregated. (Arguably, σ trended up slightly, as did the 120° ρ_*PL*_ parameter). Progressive increases that exclusively affect the location parameters are consistent with a rescaling of the distributions. The bootstrap analyses indicate that the 60° distribution is a near exact rescaled copy of the baseline 0° distribution. This implies multiplying the 0° distribution by a constant will approximate the shape of the 60° distribution.

**Figure 9:**
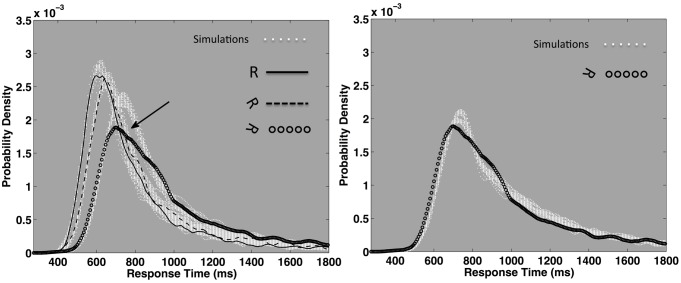
**The left-hand plot depicts the outcome of three rescaling simulations.** Synthetic cocktail response times were generated based on the fitted parameters for the baseline 0° condition (solid black line). Kernel smoothed densities for 22 replications of the synthetic distribution are depicted as white points, plotted behind the empirical 0° density. Rescaled synthetic 60° distributions were generated by first computing the natural logarithm for each synthetic baseline 0° distribution, after which, a constant of 0.061 was added to each value. Likewise a constant of 0.173 was added to duplicate the baseline synthetic data and mimic the 120° distribution. These two values represent the difference between the 0° Ω_*LN*_ parameter and the same parameter for the 60° and 120° distributions (see Table 2). The antilog of each synthetic distribution was then computed, thus yielding a rescaled model for both the empirical 60° and 120° distributions. The rescaled synthetic 60° and 120° distributions are both plotted as white points behind the empirical distributions (dashed line, and open circles, respectively). In this case, the pure multiplicative operation properly located the synthetic distributions, but the synthetic 120° distribution was more peaked at the mode than the empirical distribution (see arrow). Additional simulations revealed that a larger Ω_*PL*_ and proportion of power law samples, in addition to the multiplicative operation, were required to approximate the empirical 120° distribution, as depicted in the right-hand plot.

One interpretation of rescaling is that an increase in the rotation angle yields a less stable incarnation of the same basic dynamic organization that governs the orientation judgment in the normal condition. In a sense, increasing the rotation effectively weakens the constraints that enable participants to make the orientation judgment, leading to a proportional weakening in the dynamic interdependencies supporting the performance. Thus, increasing the rotation angle dilates the dynamics that support the act in a manner that resembles “zooming in” on a self-similar fractal object by requiring additional dynamic flow to disambiguate normal and mirror-reversed orientations, relative to the 0° baseline.

An accurate description of the 120° distribution required slight increases in Ω_*PL*_ and the proportion of power law samples, over and above a pure rescaling operation. If one assumes that discriminating orientations is more difficult when characters are increasingly rotated, then a plausible working hypothesis is that rotation progressively destabilizes this cognitive activity. Multiplicative compensation is sufficient to overcome the perturbations induced by the 60° rotation. However, less constrained interdependent power law dynamics become more likely with increased character rotation. Apparently, in this case, cognitive dynamics unfold near a point of qualitative change.

The dynamic patterns observed in these conditions unfold in a manner that is reminiscent of near-critical systems that are approaching critical points. As such, we speculate that rescaling may represent a minimum boundary of change as task difficulty, broadly construed, increases in the face of a relatively skilled performance. At some point the manipulation overwhelms the key constraints supporting the performance, and a cognitive system must either make do with ambiguous, unreliable, or strongly competing constraints, or perhaps it must reorganize and entrain with alternative reliable sources of constraint. Clearly additional research on this topic is needed, and we continue to pursue these issues in our laboratory.

## Conclusion

In a sense, this article has now come full circle. It began with an overview of the fractal geometry. The crux concept of a fractal is the notion of nesting and self-similarity—fractal objects are said to be composed of rescaled copies of the whole object. We now see that, at least for the narrowly circumscribed mental rotation data, the response time distributions can be plausibly described as rescaled copies of each other. Not all cognitive effects can be expected to fit into such a neat package. One more typically observes changes in shape representing variability increases that are larger and well beyond the limits circumscribed by a rescaling hypothesis.

Ideal mathematical fractals are typically generated through iteration—the repeated application of the same rule. This is an example of a single process that extends across multiple scales. Additional paths to scaling are available to physical and biological systems. Short-range interactions facilitate the emergence of multi-scale entrainment among rice grains in a rice pile and in so-called dynamic critical systems (e.g., Bruce and Wallace, 1989; Bak, 1996; Jensen, 1998). Model self-organizing physical systems, such as rice piles, tend to be comprised of many relatively simple homogenous elements. By contrast, complex organisms, such as human beings, entail heterogeneous physiochemical and neurophysiological processes and constraints that span a range of temporal and spatial scales. Nevertheless, these processes must somehow coordinate to support and sustain an organism across space and time. As we explained, the fractal scaling expressed in event distributions derived from biological systems, and related empirical patterns, are likely symptomatic of the dynamics governing this multiscale coordinative activity (Bassingthwaighte et al., 1994; Turvey, 2007; Holden and Rajaraman, 2012).

### Conflict of interest statement

The authors declare that the research was conducted in the absence of any commercial or financial relationships that could be construed as a potential conflict of interest.
